# Supplementary figures and images for: Integration of a physiologically-based pharmacokinetic model with a whole-body, organ-resolved genome-scale model for characterization of ethanol and acetaldehyde metabolism
Source: PLoS Comput Biol. 2021 Aug 5;17(8):e1009110. doi: 10.1371/journal.pcbi.1009110 (PMC8370625; doi:10.1371/journal.pcbi.1009110)

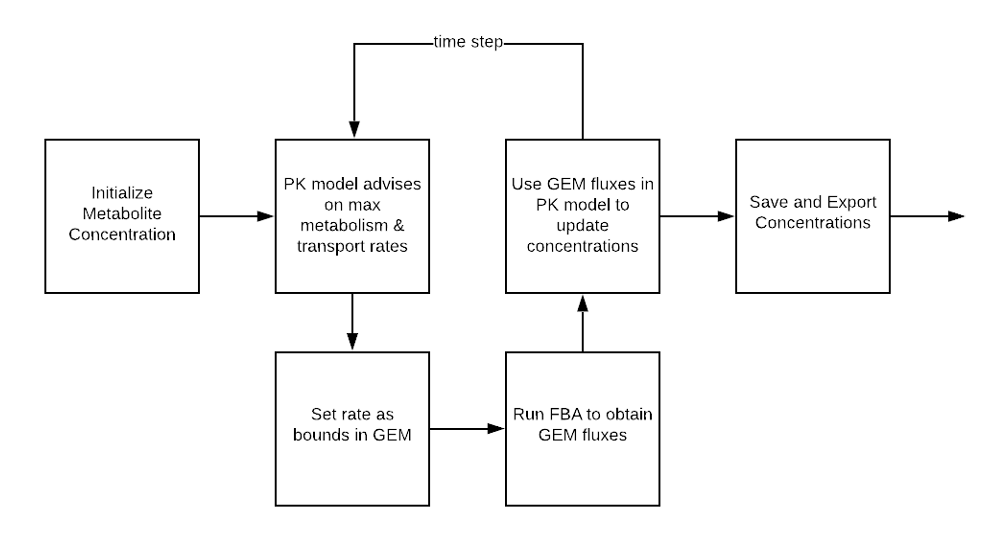

Supplement: S1 Fig — (TIF) [file pcbi.1009110.s001.tif]

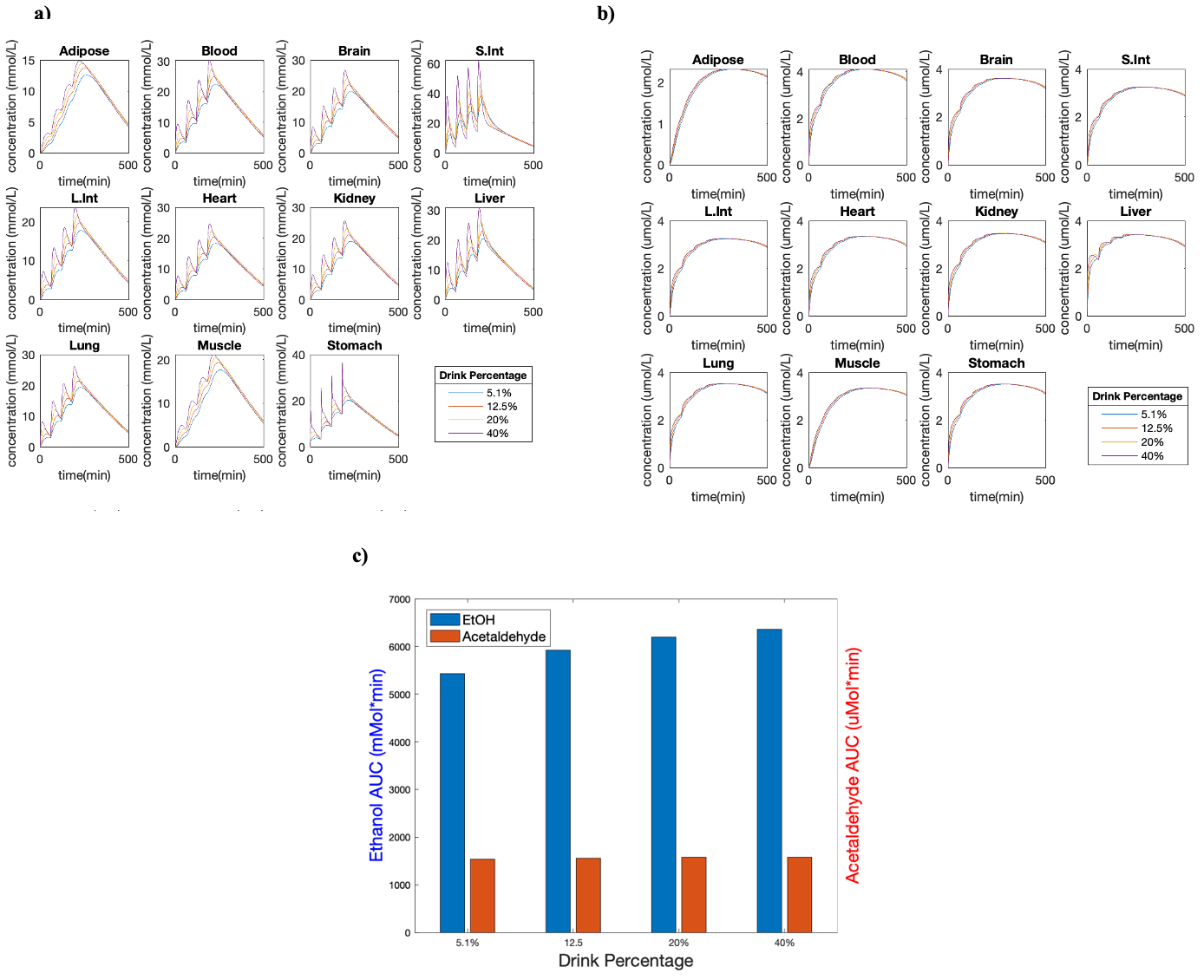

Supplement: S2 Fig — Simulations were performed with 20% ethanol for 25.6 year old men weighing 74.5kg with height = 180cm and 20% body fat drinking 0.25g/kg ethanol. (a) Multi-dosing curves for ethanol concentration in various tissue compartments. (b) Multi-dosing curves for acetaldehyde concentration in various tissue compartments. (c) Area Under the Curve for both ethanol and acetaldehyde in the liver. (TIF) [file pcbi.1009110.s002.tif]

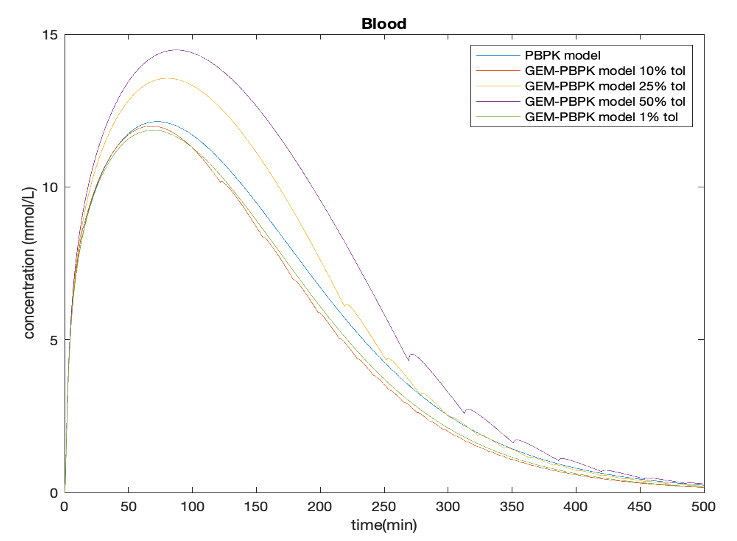

Supplement: S3 Fig — As tolerance decreases, the curve better approximates the normal PBPK model. The predictions at 1% and 10% are at lower values when compared to the PBPK model because the WBM model also predicts for methods of ethanol elimination beyond Alcohol Dehydrogenase. (TIF) [file pcbi.1009110.s003.tif]
