# Supplementary material for: Integration of a physiologically-based pharmacokinetic model with a whole-body, organ-resolved genome-scale model for characterization of ethanol and acetaldehyde metabolism
Source: PLoS Comput Biol. 2021 Aug 5;17(8):e1009110. doi: 10.1371/journal.pcbi.1009110 (PMC8370625; doi:10.1371/journal.pcbi.1009110)
Supplement: S1 Table — (DOCX) [file pcbi.1009110.s004.docx]

## S1 Table: Ethanol-related reactions

S1 Table: List of ethanol-related reactions

| Reaction # | Reaction Name | Reaction # | Reaction Name |
| --- | --- | --- | --- |
| 1482 | Alcohol Dehydrogenase, Forward Rxn (Ethanol -> Acetaldehyde) (Colon)' | 18976 | Exchange of Ethanol (from[e] toblood)' |
| 1607 | Catalase A, Peroxisomal (Ethanol) (Colon)' | 19500 | EX_etoh[u]' |
| 1884 | Transport of Ethanol, Reversible, Peroxisomal (Colon)' | 24014 | Ethanol MonooxygenaseTransport of Ethanol, Reversible, PeroxisomalEthanol Reversible Transport (BBB)' |
| 3664 | Biomass maintenance reaction without replication precursorsBiomass maintenance reaction without replication, transcription, and translation precursors (Colon)' | 30871 | Biomass maintenance reaction without replication precursorsBiomass maintenance reaction without replication, transcription, and translation precursors (Skin)' |
| 4467 | Transport of Ethanol, Reversible, PeroxisomalEthanol Reversible Transport (Colon)' | 31521 | Alcohol Dehydrogenase, Forward Rxn (Ethanol -> Acetaldehyde) (Adipocytes)' |
| 4664 | Transport of Ethanol, Reversible, PeroxisomalEthanol Reversible Transport (Colon)' | 31522 | Alcohol Dehydrogenase (Ethanol, NADP), Forward Reaction (Adipocytes)' |
| 4841 | Exchange of Ethanol (Colon)' | 32552 | Transport of Ethanol, Reversible, PeroxisomalEthanol Reversible Transport (Adipocytes)' |
| 5010 | Exchange of Ethanol (Colon)' | 32802 | Exchange of Ethanol (from[e] toblood)' |
| 5563 | Exchange of Ethanol (LI)' | 56999 | Exchange of Ethanol (Excretion)' |
| 5920 | Alcohol Dehydrogenase, Forward Rxn (Ethanol -> Acetaldehyde) (Liver)' | 57891 | Exchange of Ethanol (SI)' |
| 9099 | Transport of Ethanol, Reversible, PeroxisomalEthanol Reversible Transport (Liver)' | 58551 | Exchange of Ethanol (SI)' |
| 9508 | Transport of Ethanol, Reversible, PeroxisomalEthanol Reversible Transport (Liver)' | 60357 | Kidney_etoh(e)_[bcK]' |
| 9666 | Transport of Ethanol, Reversible, PeroxisomalEthanol Reversible Transport (Liver)' | 61125 | Exchange of Ethanol (Kidney)' |
| 9909 | Exchange of Ethanol (Liver)' | 62318 | Ethanol MonooxygenaseTransport of Ethanol, Reversible, PeroxisomalEthanol Reversible Transport (BBB)' |
| 10053 | Exchange of Ethanol (Liver)' | 63555 | Exchange of Ethanol (GI)' |
| 10617 | Exchange of Ethanol (from[e] toblood)' | 63922 | Exchange of Ethanol (Diet)' |
| 13521 | Biomass maintenance reaction without replication precursorsBiomass maintenance reaction without replication, transcription, and translation precursors (Pancreas)' | 77223 | Skin_EX_etoh(swS)_[sw]' |
| 13723 | Transport of Ethanol, Reversible, PeroxisomalEthanol Reversible Transport (Pancreas)' | 77224 | EX_etoh[sw]' |
| 14058 | Transport of Ethanol, Reversible, PeroxisomalEthanol Reversible Transport (Pancreas)' | 77225 | Skin_etoh_DIFF(c)_[swS]' |
| 14344 | Exchange of Ethanol (Pancreas)' | 77381 | Biomass maintenance reaction without replication precursorsBiomass maintenance reaction without replication, transcription, and translation precursors (Liver)' |
| 14632 | Exchange of Ethanol (frombloodto[e]' | 77386 | Biomass maintenance reaction without replication precursorsBiomass maintenance reaction without replication, transcription, and translation precursors (Adipocytes)' |
| 14711 | Exchange of Ethanol (BileDuct)' | 81095 | ‘Skin_EX_etoh(c)_[bc]’ |
| 17276 | Biomass maintenance reaction without replication precursorsBiomass maintenance reaction without replication, transcription, and translation precursors (Kidney)' | 81096 | ‘Lung_EX_etoh(br)_[bc]’ |
| 18452 | Kidney_EX_etoh(e)_[bc]' | 81097 | ‘EX_etoh[br]’ |
