# Supplementary material for: Integration of a physiologically-based pharmacokinetic model with a whole-body, organ-resolved genome-scale model for characterization of ethanol and acetaldehyde metabolism
Source: PLoS Comput Biol. 2021 Aug 5;17(8):e1009110. doi: 10.1371/journal.pcbi.1009110 (PMC8370625; doi:10.1371/journal.pcbi.1009110)
Supplement: S2 Table — (DOCX) [file pcbi.1009110.s005.docx]

## S2: Acetaldehyde-related reactions

S2 Table: List of acetaldehyde-related reactions

| **Reaction #** | **Reaction Name** | **Reaction #** | **Reaction Name** |
| --- | --- | --- | --- |
| 1393 | Acetaldehyde Mitochondrial Diffusion' | 14287 | Exchange of Acetaldehyde (Pancreas)' |
| 1394 | Acetaldehyde Peroxisomal Diffusion' | 18453 | Exchange of Acetaldehyde (frombloodto[e]' |
| 1482 | "Alcohol Dehydrogenase, Forward Rxn (Ethanol -> Acetaldehyde)"' | 24015 | Acetaldehyde Mitochondrial DiffusionAcetaldehyde Reversible Transport, Endoplasmatic ReticulumAcetaldehyde Peroxisomal DiffusionAcetaldehyde Reversible Transport (BBB)' |
| 1485 | "Aldehyde Dehydrogenase (Acetylaldehyde, NAD), Mitochondrial"' | 27019 | Exchange of Acetaldehyde (from[e] toblood)' |
| 1486 | "Aldehyde Dehydrogenase (Acetaldehyde, NADP)"' | 32803 | Exchange of Acetaldehyde (from[e] toblood)' |
| 1607 | "Catalase A, Peroxisomal (Ethanol)"' | 34199 | Exchange of Acetaldehyde (from[e] toblood)' |
| 1854 | Deoxyribose-Phosphate Aldolase' | 39992 | Exchange of Acetaldehyde (from[e] toblood)' |
| 3896 | "Aldehyde Dehydrogenase (Acetaldehyde, NAD)"' | 43159 | Exchange of Acetaldehyde (from[e] toblood)' |
| 4416 | Acetaldehyde Mitochondrial DiffusionAcetaldehyde Reversible Transport, Endoplasmatic ReticulumAcetaldehyde Peroxisomal DiffusionAcetaldehyde Reversible Transport (Colon)' | 48388 | Exchange of Acetaldehyde (from[e] toblood)' |
| 4794 | Exchange of Acetaldehyde (Colon)' | 50162 | Exchange of Acetaldehyde (from[e] toblood)' |
| 5220 | Exchange of Acetaldehyde (SI)' | 52468 | Exchange of Acetaldehyde (from[e] toblood)' |
| 5494 | Exchange of Acetaldehyde (LI)' | 53545 | Exchange of Acetaldehyde (from[e] toblood)' |
| 5836 | "Acetaldehyde Reversible Transport, Endoplasmatic Reticulum"' | 54747 | Exchange of Acetaldehyde (from[e] toblood)' |
| 5920 | "Alcohol Dehydrogenase, Forward Rxn (Ethanol -> Acetaldehyde)"' | 55819 | Exchange of Acetaldehyde (from[e] toblood)' |
| 5922 | "Aldehyde Dehydrogenase (Acetaldehyde, NADP)"' | 56659 | Exchange of Acetaldehyde (from[e] toblood)' |
| 6186 | Deoxyribose-Phosphate Aldolase' | 56857 | Exchange of Acetaldehyde (Excretion)' |
| 6644 | Cytochrome P450 17A1' | 58421 | Exchange of Acetaldehyde (SI)' |
| 7035 | Ethanolamine-Phosphate Phospho-Lyase (Deaminating)' | 60934 | Exchange of Acetaldehyde (Kidney)' |
| 9045 | "Aldehyde Dehydrogenase (Acetaldehyde, NAD)"' | 69647 | Exchange of Acetaldehyde (from[e] toblood)' |
| 9100 | Acetaldehyde Reversible Transport' | 72324 | Exchange of Acetaldehyde (GI)' |
| 10618 | Exchange of Acetaldehyde (from[e] toblood)' | 76835 | Acetaldehyde Mitochondrial DiffusionAcetaldehyde Reversible Transport, Endoplasmatic ReticulumAcetaldehyde Peroxisomal DiffusionAcetaldehyde Reversible Transport (BBB)' |
| 10754 | "Aldehyde Dehydrogenase (Acetaldehyde, NADP)"' | 80719 | Acetaldehyde Reversible Transport' |
| 10905 | Deoxyribose-Phosphate Aldolase' | 80978 | Acetaldehyde Reversible Transport' |
| 11411 | Ethanolamine-Phosphate Phospho-Lyase (Deaminating)' | 81098 | Pancreas_EX_acald[luP]' |
| 11806 | "Aldehyde Dehydrogenase (Acetaldehyde, NAD)"' |  |  |
