# Supplementary material for: Integration of a physiologically-based pharmacokinetic model with a whole-body, organ-resolved genome-scale model for characterization of ethanol and acetaldehyde metabolism
Source: PLoS Comput Biol. 2021 Aug 5;17(8):e1009110. doi: 10.1371/journal.pcbi.1009110 (PMC8370625; doi:10.1371/journal.pcbi.1009110)
Supplement: S1 Text — (DOCX) [file pcbi.1009110.s006.docx]

## S1 Text: Organ-specific equations

Tissue-specific model equations:

| $\frac{dC_{i}}{dt}=\frac{Q_{i}}{V_{i}}\left( C_{blood}-\frac{C_{i}}{K_{i}} \right)$ | S1.1 |
| --- | --- |

The equation for the arterial blood concentration is:

| $\frac{dC_{Blood}}{dt} = \frac{Q_{Lung}}{V_{Lung}} \left( \frac{C_{Lung}}{K_{Lung}} - C_{Blood} \right)$ | S1.2 |
| --- | --- |

The equations for the lungs, kidneys, and skin are shown below. The R_i_ represents the clearance rate by organ i to the outside environment.

| $\frac{dC_{Lung}}{dt} = \frac{Q_{Lung}}{V_{Lung}} \left( C_{Vein} - \frac{C_{Lung}}{K_{Lung}} \right)- R_{lung}$ | S1.3 |
| --- | --- |

| $\frac{dC_{Kid}}{dt} = \frac{Q_{Kid}}{V_{Kid}} \left( C_{Blood} - \frac{C_{Kid}}{K_{Kid}} \right)- R_{Kid}$ | S1.4 |
| --- | --- |
| $\frac{dC_{Skin}}{dt} = \frac{Q_{Skin}}{V_{Skin}} \left( C_{Blood} - \frac{C_{Skin}}{K_{Skin}} \right)- R_{Skin}$ | S1.5 |
|  |  |

The equations for the stomach, small and large intestines are shown below. The k_i_ represents the absorbance from the luminal component of organ i into the tissue. The Ri represents the clearance rate by organ i to the outside environment

| $\frac{dC_{Stom}}{dt} = \frac{Q_{Stom}}{V_{Stom}} \left( C_{Blood} - \frac{C_{Stom}}{K_{Stom}} \right)+ k_{Stom} * C_{L},_{Stom} - R_{Stom}$ | S1.6 |
| --- | --- |

| $\frac{dC_{SI}}{dt} = \frac{Q_{SI}}{V_{SI}} \left( C_{Blood} - \frac{C_{SI}}{K_{SI}} \right)+ k_{SI} * C_{L},_{SI}$ | S1.7 |
| --- | --- |

| $\frac{dC_{LI}}{dt} = \frac{Q_{LI}}{V_{LI}} \left( C_{Blood} - \frac{C_{LI}}{K_{LI}} \right)+ k_{LI} * C_{L},_{LI}- R_{Catalase}$ | S1.8 |
| --- | --- |

The equations for Q_Liv,in_ and C_Liv,in_ are shown below. The organs used for the calculations include the stomach, small and large intestines, pancreas, and spleen. Q_Liv_ represents the blood flow from the hepatic artery, Q_Liv,in_ represents the total blood flow into the liver after the mixing of the hepatic artery with the hepatic portal vein, and C_Liv,in_ represents the concentration of metabolites after the hepatic artery mixes with the hepatic portal vein.

| $Q_{Liv},_{in} = Q_{Stom} + Q_{SI} + Q_{LI} +Q_{Pancreas} + Q_{Spleen} + Q_{Liv}$ | S1.9 |
| --- | --- |

| $C_{Liv},_{in} = \frac{\sum_{i=1}^{5} \frac{Q_{i}*C_{i}}{K_{i}}+ Q_{Liv} * C_{Blood}}{Q_{Liv},_{in}}$ | S1.10 |
| --- | --- |

The equation for C_Vein_ is shown below. C_Vein_ represents the concentration of the venous blood returning to the lungs after mixing in the heart. The organs involved include adipose, brain, heart, kidney, liver (hepatic vein), muscle, and skin:

| $C_{Vein} = \frac{\sum_{i=1}^{7} \frac{Q_{i}*C_{i}}{K_{i}}}{Q_{Lung}}$ | S1.11 |
| --- | --- |

The equation for the liver is:

| $\frac{dC_{Liv}}{dt} = \frac{Q_{Liv},_{in}}{V_{Liv}} \left( C_{Liv},_{in} - \frac{C_{Liv}}{K_{Liv}} \right)- R_{Liv}$ | S1.12 |
| --- | --- |

The equations for the luminal compartments of the stomach, small intestines, and large intestines are listed below. The k_i_ represents the absorbance from the lumen into the tissue, and the k_j_ represents the transport of metabolites from the lumen of compartment I to the lumen of compartment j. In the luminal compartments, the concentration changes are affected by the absorption of metabolites through the GI tract, allowing for the model to be used in oral cannabis dosing in the future.

| $\frac{dC_{L},_{stom}}{dt}= -\left( k_{\mathrm{stom}} + k_{\mathrm{stomSI}} \right) * C_{L},_{\mathrm{stom}}$ | S1.13 |
| --- | --- |

| $\frac{dC_{L},_{SI}}{dt}= k_{stomSI} * C_{L},_{stom} - \left( k_{SI} +k_{SILI} \right)* C_{L},_{SI}$ | S1.14 |
| --- | --- |

| $\frac{dC_{L},_{LI}}{dt} = k_{SILI} * C_{L},_{SI} - \left( k_{LI} + k_{poop} \right)* C_{L},_{LI}$ | S1.15 |
| --- | --- |

The Cardiac Output is determined using the following equation where CO represents the cardiac output, BSA represents the body surface area and age is the age of the patient tested.

| $CO = (159 * BSA - 1.56 * age + 114)$ | S1.16 |
| --- | --- |
