# Supplementary material for: Integration of a physiologically-based pharmacokinetic model with a whole-body, organ-resolved genome-scale model for characterization of ethanol and acetaldehyde metabolism
Source: PLoS Comput Biol. 2021 Aug 5;17(8):e1009110. doi: 10.1371/journal.pcbi.1009110 (PMC8370625; doi:10.1371/journal.pcbi.1009110)
Supplement: S2 Text — (DOCX) [file pcbi.1009110.s007.docx]

## S2: Correlation between drink concentration and gut absorption

| $kStom = 0.7135*{Drink\%}^{2}-0.0985*Drink\%+ 0.0112$ | S2.1 |
| --- | --- |
| $kStomSI = 1.953*{Drink\%}^{2}-0.168*Drink\%+ 0.0255$ | S2.2 |
| $kSI = -0.006*{Drink\%}^{2}-0.0686*Drink\%+ 0.0615$ | S2.3 |
