# Supplementary material for: Integration of a physiologically-based pharmacokinetic model with a whole-body, organ-resolved genome-scale model for characterization of ethanol and acetaldehyde metabolism
Source: PLoS Comput Biol. 2021 Aug 5;17(8):e1009110. doi: 10.1371/journal.pcbi.1009110 (PMC8370625; doi:10.1371/journal.pcbi.1009110)
Supplement: S3 Text — (DOCX) [file pcbi.1009110.s008.docx]

## S3: Correlation between Disulfiram concentration [uM] and ALDH2 activity

| $aALDH = 0.0015*\left[ Disulfiram \right]^{2}-0.0734*[Disulfiram]+ 1$ | S3.1 |
| --- | --- |
