# Supplementary material for: Integration of a physiologically-based pharmacokinetic model with a whole-body, organ-resolved genome-scale model for characterization of ethanol and acetaldehyde metabolism
Source: PLoS Comput Biol. 2021 Aug 5;17(8):e1009110. doi: 10.1371/journal.pcbi.1009110 (PMC8370625; doi:10.1371/journal.pcbi.1009110)
Supplement: Attachment — Submitted filename: Reply to Reviewers Comments - PLoS CB.docx [file pcbi.1009110.s009.docx]

In addition to a detailed response to the reviewers' comments the revision has to address the novelty of your investigations vs the Umulis 2005 paper (mentioned by one of the reviewers), and the contribution the novel components provide to a new understanding of the metabolism of ethanol.

please upload the following:

[1] A letter containing a detailed list of your responses to the review comments and a description of the changes you have made in the manuscript.

[2] Two versions of the revised manuscript: one with either highlights or tracked changes denoting where the text has been changed; the other a clean version (uploaded as the manuscript file).

Please prepare and submit your revised manuscript within 60 days (March 29th). If you anticipate any delay, please let us know the expected resubmission date by replying to this email.

Reviewer #1: Thanks for the opportunity to review the article. While the model is generally interesting, there are some major issues with the manuscript. Here are my comments for the authors to consider.

**1. Please briefly discuss why an input in the lung is necessary in the model, shown in Figure 1.**

Apologies for the oversight, the model was adapted from a previously constructed cannabis model in which intake through the lung was considered. For the present model, there is no input of ethanol through the lungs, but there is detectable output. In the model parameters the input through the lung compartment is 0. The schematic has been modified to accurately reflect this.

**2. Please check the units of Figure 3, including Ethanol concentration and AUC in inset figure.**

Apologies for the oversight, the units have been updated to mMol*min and the concentration units have been specified.

**3. The authors established a correlation between ethanol concentration and kinetic rates. However, there is no observed data beyond this range of 5.1% to 20%. Considering the observed range is relatively narrow, extrapolating to 40 and 100% is dangerous, as there is no data to support the same relation exists beyond the 20% limit. Unless the authors could find data to support such a claim, I would suggest removing the simulations at 40% and 100%, as the simulated results could be misleading to the readers.**

Thank you for your suggestion. After consideration, we have removed the extrapolating simulations and recreated figure 3. It now only shows the data points from Mitchell et al and our predictions for 5.1%, 12.5%, and 20%.

**4. Section 3.2: while prediction of ethanol concentration from Jones et al 1988 is by-and-large ok, the prediction of early phase acetaldehyde clearly misses the observed data. The observed data indicates an abrupt increase of acetaldehyde that’s not captured by the model. However, the time to peak of acetaldehyde seems to be earlier than its parent compound, alcohol, which is a bit hard to believe. I would urge the authors to find other sources of acetaldehyde data and see if they have a better fit and validation of their model. Capturing AUC should not be used as validation to predict profiles over time. Please also discuss the limitation of predicting these profiles.**

Thank you for your suggestion. While data on ethanol concentrations in humans is ubiquitous, there is a severe lack of data on acetaldehyde (and acetate) in human studies. Prior to model simulation, possible sources of data considered for acetaldehyde concentrations are included in the table below (with their accompanying benefits and drawbacks).

Many studies observed the ratio of ethanol and acetaldehyde in the blood (taken at a specific point in time), yet this information is not usable in the current PBPK study as it lacks the kinetic information. The only studies with time-concentration data on both ethanol and acetaldehyde concentrations were Jones et al 1988, Sarkola et al 2002, Ylikahri et al 1974/1976, and Umulis et al 2005 (which used data from Jones et al 1988). The Sarkola and Ylikahri experiments were longer Jones’, yet did not record data with the same frequency (every 15 minutes), resulting in data points that did not show the early data points involving absorption and distribution kinetics of the metabolites. In addition, since the Jones study already had a PBPK model created from the data, we sought to build upon Umulis’ fitted Michaelis-Menten parameters and integrate the genome scale model constrained by those constants. Thus, after further consideration, we have decided that the data used in the Umulis Model (Jones et al 1988) is still the best data available as it captures the interaction between both ethanol and acetaldehyde, can show the pharmacokinetics of the metabolites, and there is information about the Michaelis-Menten kinetics readily available for integration. We also added in the predictions from the Umulis Model at the same time points as ours to demonstrate the comparison between models.

Regarding validation of the model, we calculated the mean average error (MAE) between our model prediction and the data from Jones et al 1988 in addition to comparing the AUC (which characterizes time-dependent exposure). While our predictions do not necessarily improve on the predictions from Umulis et al, the novelty of this work is that we successfully integrated a genome-scale model to advise on both upstream and downstream reactions pathways without altering the predictive abilities of the model, establishing a framework that can allow for future genomic studies targeted at facilitating personalized medicine. Limitations of these predictions as well as explanations for the failure of both models to predict the early peak of acetaldehyde are discussed in the newly added discussion section 4.2.

| Study | Pros | Cons | Comments |
| --- | --- | --- | --- |
| Jones et al 1988 | Time-concentration data of both ethanol and acetaldehyde, in human males, 180 minutes with 15 minute measurements | Small sample size, n=10 | Measurements not taken by blood, but by breath concentration analyzed with gas chromatography |
| Helander et al 1993 | Tracks both soluble and bound acetaldehyde in blood | Small sample size, n=4 | More focused on acetaldehyde storage rather than time-concentration data |
| Majchrowicz et al 1970 | Has figure relating acetaldehyde concentration to ethanol concentration, but only at patient intake (no control) | Only 2 graphs of time-concentration data, both n=1 |  |
| Wicht et al 1995 | Compares ethanol/acetaldehyde kinetics in patients with and without alcoholic liver disease | Data only goes to 90 minutes, small sample size n=13 |  |
| Eriksson 1982 | Compares ratio of ethanol to acetaldehyde in both men and women | No actual time-concentration data given, only correlation |  |
| Umulis et al 2005 | Minimal PBPK model with michaelis-menten constants fitted to data | Can only |  |
| Sarkola et al 2002 | Time-concentration data of both ethanol and acetaldehyde, 300 minutes with 5 measurements | Small sample size, n=13, only 5 data points on graph | Also examines the effect of oral contraceptives, lacking data points for early time analysis |
| Ylikahri et al 1976 | Time-concentration data of both ethanol , acetaldehyde, and lactate, 20 hours | Data was confounded with groups ingesting glucose/fructose, lactate shows no significant trends | This study was more aimed at studying the effects of hangover rather than elimination, not enough data at early time points |
| Ylikahri et al 1974 | Time-concentration data of ethanol and acetaldehyde, over 18 hours | Data points only collected at hours 0, 2, 4, 8, 12, 14, 16. | Not useful for seeing early kinetics |
| Roine et al 1991 | Time-concentration data of ethanol only over 240 minutes | No data on acetaldehyde, mixed sex, n=8 |  |

**5. Please discuss whether hepatic impairment will lead to changes in expression or activity of enzymes involved in alcohol metabolism.**

Thank you for your suggestion. A discussion of hepatic impairment involving inducing liver enzymes as well as cirrhosis was included in the discussion section 4.3.

**6. I do not think changing enzyme activities numerically could be directly related to a percentage change of liver functions, as suggested in Figure 5. Unlike renal impairment where a direct measurement of eGFR etc could give a good estimation of renal function, there is no such value for liver impairment, as grade of which is usually defined by a composite CP score. Please update Figure 5 where “% liver function” is used to something like “% liver enzyme activity” and the related language in text. Same goes for figure SI.3.**

Thank you for your suggestion. After reviewing the literature, we acknowledge that while there are tests available to measure specific protein production rates, it is hard to characterize the function of the liver as a whole. Scores such as the Child-Pugh and MELD scores exist to determine the extent of cirrhosis and need of transplant, however the score does not have a mathematical correlation to the amount of functioning liver. When drafting the manuscript, the % function was used with cirrhotic livers in mind, as it characterized the portion of the liver that remained functional. However, since this statistic is hard to physiologically measure, we agree that it would be more feasible to account for the inducibility of liver enzyme activity instead. A more in-depth discussion of this change is in discussion section 4.3. Figure SI.3 has been removed in favor of the new results in section 3.3.

**7. Figure 5b: the total eliminated ethanol combining all organs are clearly different for different “% liver function”. For example, more ethanol are cleared in the 100% category than that in 10% category. By a simple rule of mass balance, if the same amount of ethanal are given, then the same amount should be eliminated regardless of their liver function. Please explain why this is the case, and if there are other significant sources of elimination, they should be plotted.**

Thank you for your suggestion. At the time of submission there were constraints limiting the total simulation time for each run. As a result, the data shown in the previous Figure 5b were based on the total amount of ethanol eliminated in only the first 180 minutes, during which the less functional livers eliminated less ethanol from the body. Upon re-running the simulations to completion (allowing all of the curves to reach an ethanol concentration of 0mMol), we re-calculated the amount eliminated, the AUC_0-inf_ instead of AUC_0-180_, and further information was derived and updated in the manuscript. In the Harvey WBM, there are no other significant sources of elimination.

**8. Why is there no discussion section?**

Apologies for the confusion. The discussion section of each result was moved to a separate section and further elaborated upon to highlight the novelty of the model and its contributions to the better understanding of ethanol metabolism.

**9. Figure SI 8: unclear organ (third figure, top row).**

Apologies for the confusion. The figure has been edited to show the organ title.

Reviewer #2: This is a well written exposition of the processes the authors used to refine the PK models for ethanol ADME. I do have a few questions that could benefit from a little more discussion/disclosure in the manuscript.

**1. On the compartmental model diagram, why is ethanol being introduced into the lung? I understand that ethanol is ingested and not inhaled.**

Apologies for the oversight, the model was adapted from a previously constructed cannabis model in which intake through the lung was considered. For the present model, there is no input of ethanol through the lungs, but there is detectable output. In the model parameters the input through the lung compartment is 0. The schematic has been modified to accurately reflect this.

**2. When describing the previously published data sets used to inform and test the model, please reference the n of subjects that went into each, so the reader has a better sense of the rigor of data going into the models.**

Apologies for the confusion. The N of each study has been included for further clarity.

**3. With regards to modeling the genetic variants of ALDH2, it needs to be more clearly disclosed that these % efficiency values come from in vitro models. I still think it is OK to use these, but it reads like (or the reader assumes) the % efficiencies came from human data.**

Apologies for the confusion, the source of the data has been added into the section titles (3.4, 4.4) for better clarity.

**4. In the results discussion, there needs to be more emphasis throughout on how your findings differ or improve upon the existing models. As a modeler who is not familiar with this particular literature, it would seem to me that you are arriving upon the same "answer" as previous models, and therefore, what is the point of making a new model? Perhaps this is not the case, but whether you do or don't improve the predictiveness of any one model or if the utility is that you have a model that can predict multiple outcomes, needs to be made clearer. The utility of predictions of how different biological variables would affect ethanol/acetaldehyde exposure is clear and justified, giving a good jumping off point for someone who wants to test this in humans.**

Thank you for your feedback. The discussion of the model has been moved to a discussions section (Sections 4.x), and more emphasis on the contributions of this model to the overall PBPK/ethanol metabolism community has been added throughout the discussion section as well as in a dedicated significance section (Section 4.8).

**Reviewer #3: Overall this paper presents an updated model from previously published models from 16 years ago. While the data is acquired from the Umulis paper, the Umulis paper itself drew the data from the primarily literature and the Authors should rely on the original source data at a minimum to optimize their models. the paper is not a signficant advance as the primary updates are parametric and an increase in the number of compartments for the PBPK. The link to the genome primarily impacts the rates laws for ALDH2 and therefore this is not a significant advance for the community in PBPK or alcohol metabolism.**

Thank you for your feedback. The data used in section 3.2 is actually from the original source, Jones et al 1988, and we have included the prediction from Umulis et al 2005 as well. Apologies for the confusion.

We have added in a comparison curve to figure 4 to demonstrate how our model differs from the Umulis model, and this information is discussed in the newly-added discussion section 4.2. The discussion of the model has been moved to a discussions section, and more emphasis on the contributions of this model to the overall PBPK/ethanol metabolism community has been added in the significance section (section 4.8) as well as throughout the discussion. In addition, the genome scale model goes beyond just Alcohol Dehydrogenase & ALDH2 and includes the contribution of other processes, including the effects of urinary excretion, sweat, breathing, and colon catalase on ethanol metabolism. A significant contribution of this model is the ability to create predictions on the effects of genomic disturbances on the overall process, and this is attributed to the fact that we integrated the comprehensive steady-state calculations made by the genome scale model with the dynamics of a PK model. In doing so, PK modellers can rely on a model that provides fluxes based on the central dogma of genetics rather than creating new fitting parameters for each “clinically unmeasurable” component of their models.

Figure Files:

While revising your submission, please upload your figure files to the Preflight Analysis and Conversion Engine (PACE) digital diagnostic tool, [https://pacev2.apexcovantage.com](https://pacev2.apexcovantage.com/). PACE helps ensure that figures meet PLOS requirements. To use PACE, you must first register as a user. Then, login and navigate to the UPLOAD tab, where you will find detailed instructions on how to use the tool. If you encounter any issues or have any questions when using PACE, please email us at figures@plos.org.

Data Requirements:

Please note that, as a condition of publication, PLOS' data policy requires that you make available all data used to draw the conclusions outlined in your manuscript. Data must be deposited in an appropriate repository, included within the body of the manuscript, or uploaded as supporting information. This includes all numerical values that were used to generate graphs, histograms etc.. For an example in PLOS Biology see here: http://www.plosbiology.org/article/info%3Adoi%2F10.1371%2Fjournal.pbio.1001908#s5.

Reproducibility:

To enhance the reproducibility of your results, PLOS recommends that you deposit laboratory protocols in protocols.io, where a protocol can be assigned its own identifier (DOI) such that it can be cited independently in the future. For instructions, please see [http://journals.plos.org/compbiol/s/submission-guidelines#loc-materials-and-methods](http://journals.plos.org/plospathogens/s/submission-guidelines)
